# Efficacy of Feed Forward and Feedback Signaling for Inflations and Chest Compression Pressure During Cardiopulmonary Resuscitation in a Newborn Mannequin

**DOI:** 10.4021/jocmr865w

**Published:** 2012-07-20

**Authors:** Peter Andriessen, Sidarto Bambang Oetomo, Wei Chen, Loe MG Feijs

**Affiliations:** a Maxima Medical Centre, Department of Pediatrics, division of neonatology, P.O. Box 7777, 5500 MB, Veldhoven, The Netherlands; b Eindhoven University of Technology, Department of Industrial Design, P.O Box 513, 5600 MB Eindhoven, The Netherlands

**Keywords:** Cardiopulmonary resuscitation, Newborn, Simulation

## Abstract

**Background:**

The objective of the study was to evaluate a device that supports professionals during neonatal cardiopulmonary resuscitation (CPR). The device features a box that generates an audio-prompted rate guidance (feed forward) for inflations and compressions, and a transparent foil that is placed over the chest with marks for inter nipple line and sternum with LED’s incorporated in the foil indicating the exerted force (feedback).

**Methods:**

Ten pairs (nurse/doctor) performed CPR on a newborn resuscitation mannequin. All pairs initially performed two sessions. Thereafter two sessions were performed in similar way, after randomization in 5 pairs that used the device and 5 pairs that performed CPR without the device (controls). A rhythm score was calculated based on the number of CPR cycles that were performed correctly.

**Results:**

The rhythm score with the device improved from 85 ± 14 to 99 ± 2% (P < 0.05). In the control group no differences were observed. The recorded pressures with the device increased from 3.1 ± 1.6 to 4.9 ± 0.8 arbitrary units (P < 0.05). The second performance of the teams showed significant better results for the group with the CPR device compared to the controls.

**Conclusion:**

Feed forward and feedback signaling leads to a more constant rhythm and chest compression pressure during CPR.

## Introduction

Approximately 10% of all newborns require assistance to start breathing at birth and in 1% of all newborns extensive resuscitative measures are warranted [1]. Actions that have to be undertaken are clearly described in the Neonatal Resuscitation Guidelines of the American Heart Association and International Liaison Committee on Resuscitation: when apnea persists and the heart rate is below 60 beats per minute then actual cardiopulmonary resuscitation (CPR) should be instituted [2]. The procedure consists of inflations of the lung and compressions of the chest. CPR should be carried out with two persons: one who is performing the inflations and one the chest compressions. The ratio of inflations and chest compressions should be 1:3 with 30 inflations and 90 compressions yielding 30 cycles per minute. Compressions should be delivered by two thumbs on the lower third of the sternum while the hands of the rescuer encircle the thorax. The recommended location for chest compression during CPR is at the lower third of the sternum.

Chest compressions can be life saving but hold the risk of inducing chest injuries. In adults sternal and rib fractures occur relative frequently but in infants chest injuries due to CPR are very rare [3-4]. During the stress of an acute situation it is difficult to exert the compressions at the right spot at a constant pressure and to maintain the right rhythm and the correct ratio of inflations to compressions [5-6]. In order to assist neonatal resuscitation we designed a device, called “Rhythm of life aid” (ROLA) that supports health care providers during CPR of the newborn. In a previous paper we have described in detail the design and technical aspects of the device [7]. The specific research questions that we asked for this study were: (a) Does the use of ROLA lead to consistent CPR with respect to the rhythm and 1:3 ratio of inflations and compressions? (b) Does the use of ROLA lead to more consistent pressure of the chest compressions?

## Methods

**Figure 1:**
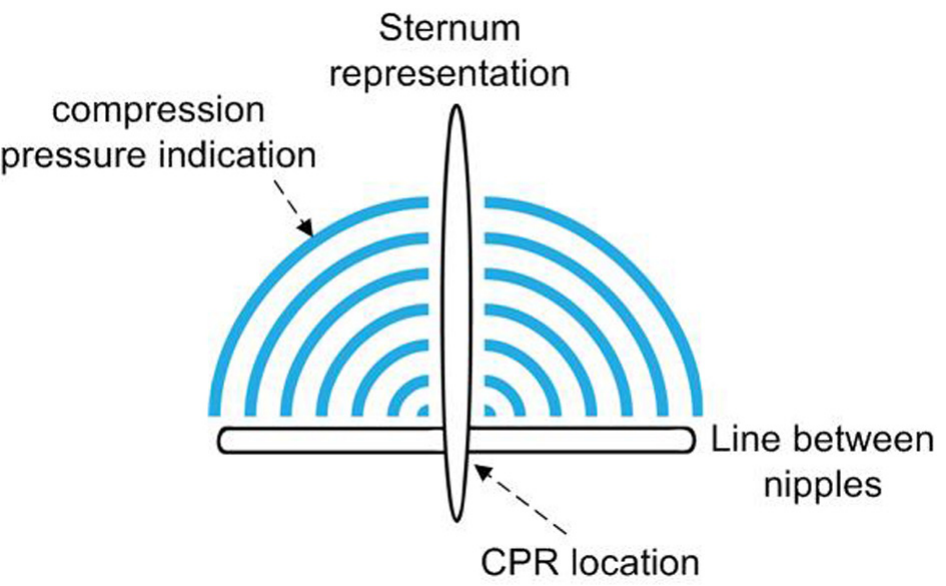
Design of the visual actuator. A force sensitive resistor is integrated as a pressure sensor in the foil at the intersection of marking of sternum and inter nipple line. The visual indicator consists of an embedded set of six parallel semi-annular electroluminescent strips that light up with increasing pressure.

**Figure 2:**
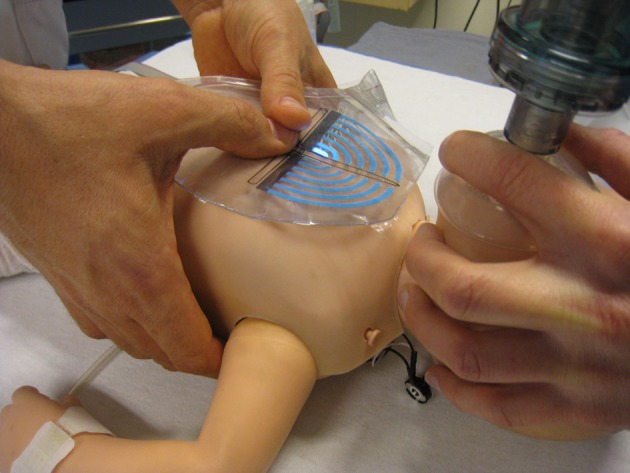
The ROLA design placed over the thorax of a newborn mannequin. The design features clear recognizable marks to be placed over sternum and inter nipple line in order to position the point of chest compressions exactly in the middle of the lower sternum. The effectiveness of external chest compression was measured with a visual indicator that lighted up with increasing pressure. The indicator was calibrated in such a way that compression of the chest up to one third of the diameter of the chest resulted in lighting of five electroluminescent strips.

The ROLA device features a box that generates an audio-prompted rate guidance with distinct sounds for inflations and compressions (feed forward), and a transparent sheet that was integrated with a visual actuator that lights up as the pressure on the foil is increased (feedback) [7]. The design features clear recognizable marks to be placed over sternum and inter nipple line in order to position the point of chest compressions exactly in the middle of the lower sternum (Fig. 1, 2). A force sensitive resistor is integrated as a pressure sensor in the foil at the intersection of the marking of the sternum and inter nipple line. The visual indicator consists of an embedded set of six parallel, semi-annular electroluminescent strips that light up with increasing pressure. The indicator was calibrated in such a way that compression of the chest up to one third of the diameter of the chest resulted in lighting of the first five electroluminescent strips. We measured a pressure of five arbitrary units when five strips lit up.

The local ethical committee approved the conduction of a simulation study on an instrumented mannequin. Ten teams consisting of a doctor and a nurse performed CPR on a instrumented newborn resuscitation mannequin, (Resusci Baby, Laerdal, Stavanger, Norway) equipped with a pressure sensor at the lower sternum. All teams initially performed two CPR sessions of 90 s: first inflation by the doctor and compressions by the nurse and second: inflation by the nurse and compressions by the doctor. Thereafter another two CPR sessions of 90 s were performed in similar way, after randomization in 5 teams that used the ROLA and 5 teams that performed CPR without the ROLA (control group). The nurses and doctors were recruited from the NICU staff. All were familiar with CPR and received training in CPR of newborns before starting their employment on the NICU. Therefore the performing of CPR in this study could be carried out without any prior instructions. The group that performed CPR with the ROLA was instructed on the features of the device.

The CPR sessions were analyzed by an investigator who was unaware of the use of the ROLA. A score was calculated based on the number of CPR cycles that were performed correctly. When CPR was carried out perfectly during 90 s, there were 45 cycles of 3 compressions and 1 inflation. The score was expressed as percentage of the cycles that have been performed correctly: (number of perfect cycles/45) x 100%.

The chest compression pressure was measured with a pressure transducer and data are expressed as arbitrary units.

Statistical analysis: the data are expressed as mean ± standard deviation. The Wilcoxon signed rank test was applied to test differences between the first and second CPR session within the groups. The Mann-Whitney U test was applied to test differences between the ROLA and the control group with respect to the parameters described above.

## Results

No significant differences were noted in the age or years of neonatal care experience between the ROLA team and control team (age, 42 ± 10 vs. 38 ± 13 years; neonatal care experience, 11 ± 9 vs. 11 ± 12 years).

Data are shown for the two sessions: the ROLA group used the ROLA device in the second session. The control group performed two sessions without the ROLA device. NS refers to non significant (Table 1).

**Table 1:** Rhythm Score Values

	First session	Second session	P-value
Controls (n = 10)	91 ± 15	90 ± 10	NS
ROLA (n = 10)	85 ± 14	99 ± 2	< 0.05
P-value	NS	< 0.01	

Legend: Rhythm scores (expressed in percentage, mean ± SD) of nurses and doctors that performed chest compressions and lung inflations during 90 s on a newborn manikin in a ratio of 3:1.

The recorded pressures in the first and second session were similar for the control group. In contrast, the recorded pressure of the ROLA group was significantly higher during the use of the ROLA. The standard deviation of the session with a ROLA device was lower than the control session indicating less variability in chest compression (Table 2). Comparison of the second performance of the teams showed significantly better results for the ROLA groups in terms of rhythm (P < 0.05) and pressure (P < 0.05).

**Table 2:** Compression Pressure Values

	First Session	Second Session	P-value
Controls (n = 10)	3.3 ± 1.0	2.4 ± 1.4	NS
ROLA (n = 10)	3.1 ± 1.6	4.9 ± 0.8	< 0.05
P-value	NS	< 0.001	

Legend: Compression pressures (expressed in arbitrary units, mean ± SD) measured during 90 s of chest compressions and lung inflations by nurses and doctors on a newborn manikin in a ratio of 3:1. Data are shown for the two sessions. The ROLA group used the ROLA device in the second session. The control group performed two sessions without the ROLA device. NS refers to non significant.

## Discussion

The ROLA device was designed to assist the health care providers during neonatal resuscitation by providing an easy portable device to find the correct location of chest compression rapidly, with the feature to indicate the depth of chest compressions. Furthermore, the design requirements of the device were feedback guidance on correct rhythm of chest compressions and ventilation for *neonatal CPR*, e.g. a compression-ventilation ratio of 3:1 at 120 beats per minute [2]. The main finding of this study is that use of the ROLA device lead to a more constant rhythm and chest compression pressure values during neonatal CPR.

The effect of visual feedback or audio-prompted rate guidance during chest compressions on the performance of CPR has been studied in adult mannequins. Visual feedback significantly improved the chest compression quality (compression rate and depth) in simulated cardiac arrest [8]. In another adult simulation study metronome guidance corrected chest compression rates for each compression cycle, but did not affect chest compression quality [9].

Limited information of feedback guidance is available in pediatrics. The effect of audio-prompted rate guidance during chest compressions on the performance of CPR has been studied earlier in a prospective study of intubated children with cardiac arrest [10]. After placement of a capnometer between the endotracheal tube and resuscitation bag, an audiotape instructed the resuscitator to perform chest compressions at 100 per min or 140 per min for one min, followed by another min at the other rate. End-tidal carbon dioxide partial pressure was recorded before the audiotape instruction and after one min of CPR at each rate. The main finding was that despite clear instruction, CPR is performed poorly and at inappropriately slow rates. The audio-prompted rate guidance during CPR in children resulted in higher end-tidal carbon dioxide, suggesting improved CPR performance [10].

To assist the learning process a feed forward and feedback was incorporated into the design [11]. Audio guidance similar to a metronome provided at a fixed rate of 120 beats per minute, starting with a (low pitch) distinct sound for inflations followed by three (high pitch) sounds for chest compression. The audio feed forward supported the CPR provider to perform the correct rhythm and ratio of compression and ventilation, respectively. A transparent foil integrated with pressure sensor and annular electroluminescent actuators with different radius formed the feedback loop: the CPR provider obtained instantaneous access to the information of chest compression pressure/depth. We hypothesize that the results in the ROLA group are attributed through a self-regulating process, where the CPR provider was able to reflect on his or her performance immediately and adjust accordingly. The lack of feed forward and feedback in the control group explains possibly that no improvement was observed between the first and second session i.e. there was no learning effect by repeated CPR performance. This study suggests that the use of the ROLA leads to better CPR performance and not a practice session of 90 s of CPR that preceded the second session of 90 s CPR.

Neonatal resuscitation is a team activity that involves at least two people working together to achieve a shared goal [12]. The quality of neonatal resuscitation is dependent on the quality of communication and the performance of specific skills. As the age and years of experience of the health care providers (nurses and doctors) were comparable, we consider the aspect less likely to explain the differences in our study. The ROLA device may serve as a tool for supporting the technical skills during neonatal resuscitation while adequate communication between professionals can proceed.

This study is limited in that it is a mannequin study in an artificial laboratory setting without measurements of output variables. Furthermore, several methodological considerations may be related to the design of the ROLA device. The location of chest compression, two-thumb compression method and calibration of the device when 1/3 of chest diameter is compressed were based on consensus of current basic life support guidelines [2]. There is a paucity of information in the literature describing these aspects of CPR in newborns. First, anthropometric measurements of the infant chest to guide the development of neonatal-specific CPR are largely unknown [13]. The optimum location for chest compressions during CPR may be lower than presumed. Evidence from multidirectional computed tomography images of infants showed that the left ventricle was located in the lower quarter of the sternum [14]. Second, in an infant model of prolonged CPR the two-thumb external compression technique produced higher blood pressure values than the two-finger technique. Rescuers found the two-thumb technique easier to perform and less fatiguing than the two-finger method [15]. Third, using computed tomography reconstruction estimates of chest dimensions indicated a chest compression of approximately one-third of the anterior-posterior chest depth radiographically is appropriate [16-17]. As there was no difference in compression depths measured at inter nipple line versus in the lower half of the sternum, we choose for the ROLA design with clear recognizable marks over sternum and inter nipple line.

### Conclusion

The use of the ROLA device leads to a more constant rhythm and pressure of chest compressions during CPR of newborn infants. Feed forward and feedback simulation strategies may improve health care providers’ ability to perform neonatal CPR. Efforts need to be made in order to improve CPR quality and to support professionals by real-time feedback. However, the technical elements of CPR feedback (e.g. depth of external compression and pressure calibration) need to be carefully studied in a neonatal animal study before being introduced into clinical practice.
